# Autistic traits relate to reduced reward sensitivity in learning from point-light displays (PLDs)

**DOI:** 10.1098/rsos.241349

**Published:** 2025-03-26

**Authors:** Raimund Buehler, Libor Potocar, Nace Mikus, Giorgia Silani

**Affiliations:** ^1^ Department of Clinical and Health Psychology, University of Vienna, Vienna, Austria; ^2^ Department of Psychology, University of Oslo, Oslo, Norway; ^3^ Department of Cognition, Emotion, and Methods in Psychology, University of Vienna, Vienna, Austria

**Keywords:** autistic traits, autism spectrum disorder, reinforcement learning, social motivation, biological motion, predictive coding

## Abstract

A number of studies have linked autistic traits to difficulties in learning from social (versus non-social) stimuli. However, these stimuli are often difficult to match on low-level visual properties, which is especially important given the impact of autistic traits on sensory processing. Additionally, studies often fail to account for dissociable aspects of the learning process in the specification of model parameters (learning rates and reward sensitivity). Here, we investigate whether learning deficits in individuals with high autistic traits exhibit deficits when learning from facial point-light displays (PLDs) depicting emotional expressions. Social and non-social stimuli were created from random arrangements of the same number of point-lights and carefully matched on low-level visual properties. Neurotypical participants (*N* = 63) were assessed using the autism spectrum quotient (AQ) and completed a total of 96 trials in a reinforcement learning task. Although linear multi-level modelling did not indicate learning deficits, pre-registered computational modelling using a Rescorla–Wagner framework revealed that higher autistic traits were associated with reduced reward sensitivity in the win domain, demonstrating an attenuated response to received feedback during learning. These findings suggest that autistic traits can significantly impact learning from PLD feedback beyond a general deficit in learning rates.

## 1. Introduction

Autism spectrum disorder (ASD) represents a wide range of neuro-developmental conditions characterized by deficits in social communication and interaction, and repetitive or restricted patterns of behaviours, interests or activities. In line with the notion of universally accepted heterogeneity of autism [1,2], the diagnostic and statistical manual of mental disorders V (DSM-V) currently adopts a dimensional approach to ASD [3], highlighting that ASD represents a spectrum extending into the general population.

While several theories have been put forward to explain ASD symptoms, and relatedly autistic traits in the general population, one of the most promising approaches to its aetiology is to describe the condition as a learning deficit [4]. For example, predictive processing construes ASD symptoms as a consequence of a core deficit in learning about the contingencies of the environment, often resulting in imprecise predictions. This is especially relevant when stimuli are complex and dynamic, i.e. when dealing with social situations [4]. Difficulties in social domains are thus often interpreted to result from a failure to adapt *learning rates* (the weighting of prediction errors) to different contexts, i.e. by overestimating the volatility of the environment and assigning excessive weight to recent information [5]. This can in turn result in difficulties with learning and behaviour in social interactions; however, supposed deficits are not necessarily confined to social situations but rather described as a general learning deficit [5,6].

A related but alternative account is the *slow updating hypothesis*, which suggests that autistic individuals integrate new information more slowly [7,8]. For example, Lieder and colleagues [7] found that autistic participants underweighted recent sensory input in a perceptual discrimination task, while Vishne and colleagues [8] showed reduced trial-by-trial error correction in a sensorimotor synchronization task. Both studies indicate that rather than an increased reliance on prediction errors, autistic learning difficulties may stem from slower short-term adjustments, potentially affecting adaptation in both social and non-social contexts.

A competing account of the learning difficulties in ASD arises from the *social motivation hypothesis* [9]. This theory proposes that ASD can be characterized by a fundamental difference in the *processing of social rewards*, similarly leading to less optimal learning in social contexts, but due to slightly different reasons [10,11]; for a review see [12]. According to this perspective, learning deficits mainly emerge as a consequence of attenuated sensitivity to rewarding social stimuli. Learning difficulties are therefore confined to social contexts, by contrast to the notion of a general learning deficit described above.

Hypotheses derived from both accounts are often tested using reinforcement learning models [5,6,13]. In these models, choice behaviour in response to rewarding feedback is investigated in a trial-by-trial approach. Different parameters are then estimated on the subject level and compared between groups. These parameters permit making conclusions about the underlying causes of learning deficits. For instance, studies often tend to focus on group differences in the learning rate *α* [14]. This parameter captures the weighting of prediction errors and measures the general *speed* at which rewards affect behaviour during learning. However, reinforcement learning models can also be specified with additional parameters, such as the reward sensitivity *ρ*. This parameter measures the *internal value* of a specific type of reward [15]. Distinguishing these components in the investigation of learning deficits is important, as they can be used to make inferences about underlying cognitive processes. For example, attempts to disentangle the contribution of each parameter have been made in computational models of depression, where anhedonia was shown to affect reward sensitivity, whereas dopamine manipulation affected learning rates [15]. Thus, both parameters are theoretically distinguishable and can be used to inform theoretical assumptions. In the context of autistic traits, reduced reward sensitivity in the social condition should reflect lower internal values for social stimuli, in line with theoretical accounts of attenuated social motivation. By contrast, differences in learning rates extending to non-social stimuli would convey a more general learning deficit, which may be particularly relevant in social situations, but not necessarily confined to them.

To test the specificity of learning difficulties in ASD, studies have so far often relied on pictures or videos of emotional expressions, which are then compared with non-social stimuli such as money or objects. While some studies found support for the domain-specific hypothesis of an impairment exclusively for social contexts [10,16–19], other results suggest deficits also in non-social conditions [11,20]. Importantly, these deficits seem to generalize to autistic traits in the general population as measured by the autism spectrum quotient (AQ) [21,22], both in regard to a general learning deficit [23–25] as well as an impairment confined exclusively to social contexts [26–28].

A major limitation and confounding aspect in these studies concerns the matching of social and non-social stimuli on low-level visual properties, which may contribute to the discrepant results about the specificity of learning deficits. Visual stimuli used for the non-social condition such as objects or shapes [17,29,30] are difficult to match in aspects like colour, texture and luminance. This can potentially impact participants’ responses [31], especially in autistic individuals for which atypicalities often arise at the sensory-perceptual level [32]. Therefore, matching stimuli on low-level visual properties is critical to properly interpret emerging differences. For this study, we have thus created a novel set of stimuli by drawing on the field of *biological motion*. This line of research has established the use of point-light displays (PLDs), a class of low-level perceptual stimuli consisting of white points on a black background, which offer separation of social and emotional content from other visual features of the stimulus. It has been demonstrated that facial emotional expressions can be recognized from PLDs [33–35] and that such stimuli can reliably activate brain regions associated with face processing, such as the fusiform face area (FFA) [36]. Furthermore, neurotypical children exhibit visual preference for PLDs depicting biological motion when compared with autistic children [37,38], and high autistic traits relate to impaired emotion recognition from facial PLDs [39]. Importantly, some researchers have connected deficits in emotion recognition to social motivation and reward processing [40].

To our knowledge, PLDs have so far not been used to investigate learning from social and non-social stimuli. In this study, we therefore examined differences in learning related to autistic traits using PLDs in a reinforcement learning task. In the social condition, participants were presented with facial PLDs depicting positive or negative emotions (i.e. happiness or anger) to learn an underlying association. In the non-social condition, initially random arrangements of the same number of point-lights formed conventional symbols of success or failure (a check mark or a cross). Our goal was to investigate how autistic traits would impact learning from social and non-social PLDs. Additionally, we were interested in whether variation in parameters of the reinforcement learning model (learning rate and reward sensitivity) in different conditions (non-social versus social) could lend support to competing theoretical accounts of learning deficits in autism (e.g. general learning deficit or attenuated sensitivity to social rewards). We expected participants with high autistic traits to perform worse in the social condition and no differences in the non-social condition, but we did not make predictions as to which parameter of the reinforcement learning model precisely was affected.

## 2. Methods

### 2.1. Sample

The sample consisted of typically developing (TD) individuals, who were contacted via a participation recruitment platform of the University of Vienna. Participants were required to fulfil the following inclusion criteria: (i) age between 18 and 65 years, (ii) heterosexual orientation, (iii) proficiency in English, (iv) no drug or alcohol addiction or regular drug use, and (v) no psychiatric or neurological condition. As the experiment was part of a larger project, some of the exclusion criteria are related to another task and not directly relevant for this study.

From the total of 74 recruited individuals, three participants were excluded due to missing data for AQ scores and five participants were dropped based on exclusion criteria. Three individuals were excluded from analyses because of missing data for the task, leading to a final sample of *N* = 63 participants for analysis. Participants received either a financial compensation of €10 or four study credits.

### 2.2. Measures—autism spectrum quotient

To measure autistic traits, a German shortened version of the autism spectrum quotient (AQ-k; [22]), widely used in research and clinical practice, was used. AQ-k contains 33 items (e.g. ‘*I prefer to do things on my own rather than with others*.’) and is suitable for adults and adolescents aged 16 years and above with normal intellectual functioning. For screening purposes of ASD in clinical practice, a cut-off of 17 was proposed [22]. In the present study, item responses were scored using a binary system, where endorsement of an autistic trait is scored with one point, while the opposite response is scored with zero, resulting in a maximum score of 33 [41]. In the present sample, we report reliability scores as Cronbach’s alpha (*α*) = 0.83 and McDonald’s omega (*ω*) = 0.85, indicating a good scale reliability.

### 2.3. Social reinforcement learning task

**Figure 1.**
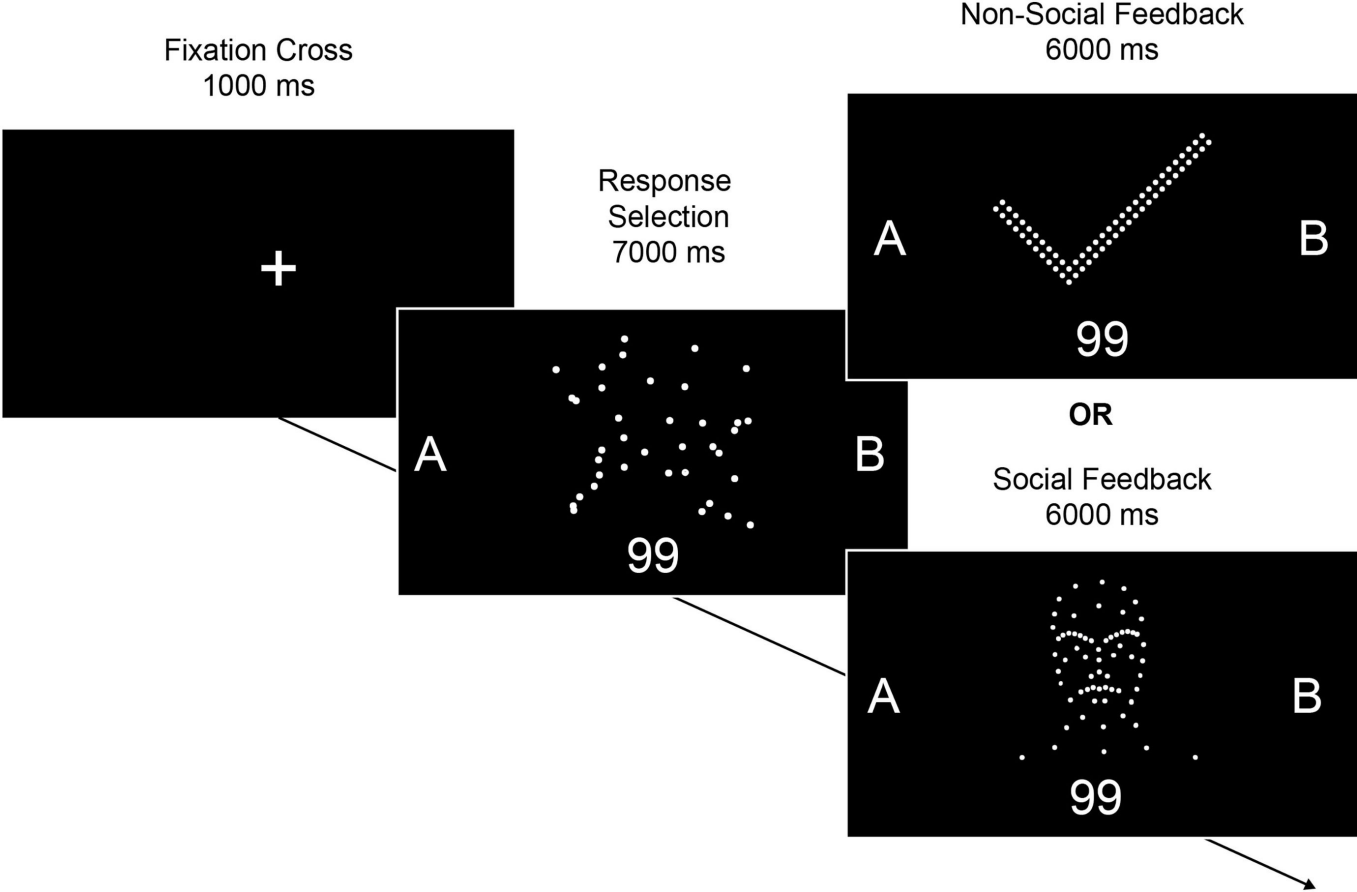
Illustration of a single trial. A random arrangement of point lights was displayed during response selection and subsequently transformed into either social or non-social feedback stimuli. Participants were asked to categorize a random number stimulus into one of two arbitrary groups, A or B. Non-social feedback consisted of videos of check marks and crosses (not displayed), while social feedback featured point-light faces expressing happy or angry emotions. Feedback was delivered probabilistically based on a reward contingency of 0.85/0.15. Each block consisted of six trials, with two social and two non-social blocks, for a total of 12 trials. Blocks were presented in an interleaved order, with the starting block type randomly selected. Note: display brightness has been enhanced for visibility.

To assess learning, we used a social reinforcement learning task with PLDs as feedback (see figure 1). Participants were required to categorize randomly generated two-digit numbers (e.g. 99) into arbitrary groups (‘A’ or ‘B’) via button press. Importantly, feedback was delivered probabilistically: in 85% of trials, correct responses were followed by rewarding feedback, while in 15% of trials correct responses were followed by non-rewarding feedback. This contingency was chosen to provide an appropriate level of difficulty for learning the underlying associations. Participants were informed that categories were arbitrary with no underlying rule, requiring them to learn via trial-and-error from feedback. The exact contingencies were not disclosed.

After each response, participants received PLD feedback. First, a random pattern of point lights was displayed, which transformed into either social or non-social feedback. In social blocks, point lights formed happy or angry human faces to indicate correct or incorrect responses. In non-social blocks, they formed check marks or crosses. Participants completed two blocks for each condition, each block consisting of six trials. Within each trial, four unique two-digit numbers were presented and repeated across subsequent trials, with the presentation order randomized. To avoid carry-over effects, even numbers were assigned to the social blocks, odd numbers to the non-social blocks. Response options (‘A’ and ‘B’) were displayed to the left and right of the screen, with their positions randomly switched between trials to avoid simple motor learning.

To control for order effects, the first block type (social/non-social) was randomly selected, and subsequent blocks alternated between the two types. The dependent variable for the GLMM analysis was the proportion of correct answers, defined as the proportion of trials in which participants chose the high probability option. We expected participants to perform around chance level (50%) in the first trial and improve in subsequent trials, as they learned the underlying reward contingencies. A German version of the task is available online (Chrome recommended): https://raimund-buehler.github.io/SOCIALRL_PLD/.

### 2.4. Statistical analysis

#### 2.4.1. Generalized linear mixed model

We used a generalized linear mixed model (GLMM) to analyse learning performance. This approach accounts for repeated-measures and dependencies in the data, such as blocks and trials nested within individuals, by incorporating a random intercept for each individual [42]. The model includes both fixed effects, which capture overall trends in the sample (e.g. the average learning trajectory) and random effects, which capture between-person variability around these average relationships (e.g. individual differences in learning trajectories). This design allows us to examine individual learning trajectories and differences in performance across blocks. Specifically, we investigated the effect of AQ scores on participants’ learning trajectories across block types, exploring whether autistic traits are associated with lower learning performance and whether this relationship occurs only in social blocks or across both block types.

Given the binary nature of the dependent variable, participant choice (correct/incorrect), a binomial distribution and logit link function was assumed. The following predictors were included in the model as fixed effects: AQ score (continuous), block type (binary: social/non-social) and trial number (continuous, 1 to 12). We performed grand-mean centring for AQ score by subtracting the overall sample mean from each individual’s score [43]. This was done to improve model convergence and ensuring more stable parameter estimates.

To assess the presence of interindividual variability in trials and block types, we performed a likelihood ratio test, to evaluate whether the inclusion of random slopes significantly improved model fit. First, we compared a model with random slopes to a simpler model that included only random intercepts by subject and fixed effects, but no random slopes. The likelihood ratio test was significant (*χ*^2^(5) = 160, *p* < 0.001), confirming significant interindividual variance in learning trajectories and block types. To examine whether AQ scores accounted for this variability in learning trajectories, we added an interaction term, yielding the following model, with AQ specified as fixed effect, trial number and block type specified as both fixed and random effects. Specifically, in pseudo-code,


(2.1) $$\begin{array}{r} Choice∼Block\;Type\times Trial\;Number\times AQ\;Score+\\ (1+Block\;Type+Trial\;Number|Participant). \end{array}$$

All model estimates are reported as odds ratios (OR) incl. 95% confidence intervals (CIs) calculated using the Wald method. Odds ratios range from 0 to ∞, with values greater than 1 indicating increased odds of giving a correct answer and values less than 1 reflecting decreased odds. Confidence intervals that include 1 suggest no significant association, whereas intervals excluding 1 indicate a statistically significant effect. All models were fitted via the maximum likelihood estimation method using the lme4 package (version 1.1−31) [44].

#### 2.4.2. Reinforcement learning model

Probabilistic reinforcement learning tasks lend themselves to a trial-by-trial analysis of responses via reinforcement learning models, which are characterized by a set of parameters [13]. These parameters capture certain aspects of the individual learning process, i.e. the ability to quickly shift behaviour in response to feedback (*learning rate*), the impact of reward reception (*reward sensitivity*) or the tendency to explore choice options under uncertainty (*temperature*). They in turn rely on the prediction error, a central component of reinforcement learning, which signals reward reception, but decays as reward contingencies grow to be expected.

We used a Rescorla–Wagner reinforcement learning model to analyse choice behaviour in a trial-by-trial approach. Such models use a set of computational steps to predict choice probabilities given the current expected probability of receiving a reward and the choice outcome on the current trial. The first step consists of computing a reward prediction error (RPE) for a given trial *t*,


(2.2) $$RPE(t)=\rho \cdot Outcome(t)-P_{A}(t),$$

with *P* denoting the estimated probability of receiving a reward and *A* denoting the choice option. The outcome is coded as a binary variable (0 for no reward and 1 for reward reception) and is scaled by the *reward sensitivity parameter ρ*. The RPE takes large values if a reward is received (positive) or omitted (negative) unexpectedly. Each trial, the expectation of receiving a reward on the next trial is updated by scaling the RPE by the *learning rate parameter* α,


(2.3) $$P_{A}(t+1)=P_{A}(t)+\alpha \cdot RPE(t).$$

Here, the RPE is used to compute the expected reward probability when choosing option A on the next trial. If a reward was received unexpectedly, the RPE will take a large positive value and the expected probability of receiving a reward on the next trial will increase. Likewise, if a reward was omitted, the RPE will be negative and the expected probability of receiving a reward on the next trial will decrease. The result of this computation is scaled by the learning rate α, which is bounded between 0 and 1 and can be estimated for each participant. Lastly, the probability of choosing the option is modelled using the softmax rule (a sigmoid function) and the *inverse temperature parameter* θ,


(2.4) $$P_{Choice\;A}(t+1)=\frac{1}{1+e^{-\theta (P_{A}-P_{B})}}.$$

The crucial point during this step is the scaling of the difference between expected probabilities for choices A or B. If θ is large, even a small difference in expected reward probabilities will bias choice behaviour towards A or B. Conversely, if it is small, the difference between probabilities needs to be very pronounced for choice behaviour to be affected. This in turn results in more or less exploration of choice options, with more deterministic choice behaviour for large values of θ and more probabilistic behaviour for low values.

This set of equations was used to model individual choice behaviour and estimate the described parameters using a set of custom python scripts. Parameters were estimated using maximum likelihood (ML), specifically by minimizing the negative likelihood using the *minimize* function from the *scipy* package with the truncated Newton conjugate (TNC) method [45]. Parameters were initialized randomly with bounds manually constrained to −10 to 10 for *α* and *ρ* and 1 to 50 for *θ*. To ensure that parameters fell within the conventional bounds of 0 and 1, a logistic transformation was applied to *α* and *ρ*. For *θ*, no logistic transformation was applied. To further improve model fit, prevent extreme values and stabilize parameter estimation, we used elastic net regularization with L1 and L2 penalties with regularization strength set to *λ* = 0.01 for both penalties. This resulted in stable estimations over several cycles of parameter fitting. Parameters were estimated separately for wins and losses as well as social and non-social conditions, resulting in a set of six parameters for each participant.

As evident from histograms, scatterplots, as well as the fact that *α* and *ρ* are bounded between 0 and 1, assumptions for linear models may be violated (see below). Indeed, quantile–quantile plots (QQplots) for linear models confirm deviation from normal distribution, mostly at the tails (see electronic supplementary material, figure S1). This suggests more extreme values in the observed data than expected under the assumption of normal distribution. Thus, for *α* and *ρ*, a GLMM with a beta distribution was considered an appropriate choice for analysis, as the beta distribution is defined on the interval between 0 and 1 and therefore naturally suitable to model data that is bounded within this range. *θ*, which was not bounded between 0 and 1, was investigated using a classical LMM approach. Diagnostic plots with the DHARMa package still indicated some assumption problems for the *ρ*_win_ model in the beta-GLMM approach; however, points aligned more closely to the diagonal line in the QQplot than for LMM models (see electronic supplementary material, figures S1 and S2).

All statistical analyses were performed using R 4.2.2 [46]. LMMs and GLMMs were fit via maximum likelihood using the *lme4* package version 1.1.31 [44]. Beta-GLMMs were fit using the *glmmTMB* package [47]. Assumptions for beta GLMMs were investigated using the *DHARMa* package version 0.4.6 (see electronic supplementary material, figure S2). Confidence intervals for odds ratios were computed using the Wald method.

In line with open science practices, Python and R scripts used for data pre-processing and statistical analysis are available online: https://github.com/raimund-buehler/RALT_final.nosync.

The statistical analysis was pre-registered at the Open Science Framework (OSF): https://osf.io/tgcqm.

A description of deviations from the pre-registered analysis is provided in the supplementary material.

## 3. Results

### 3.1. Sample characteristics

**Figure 2.**
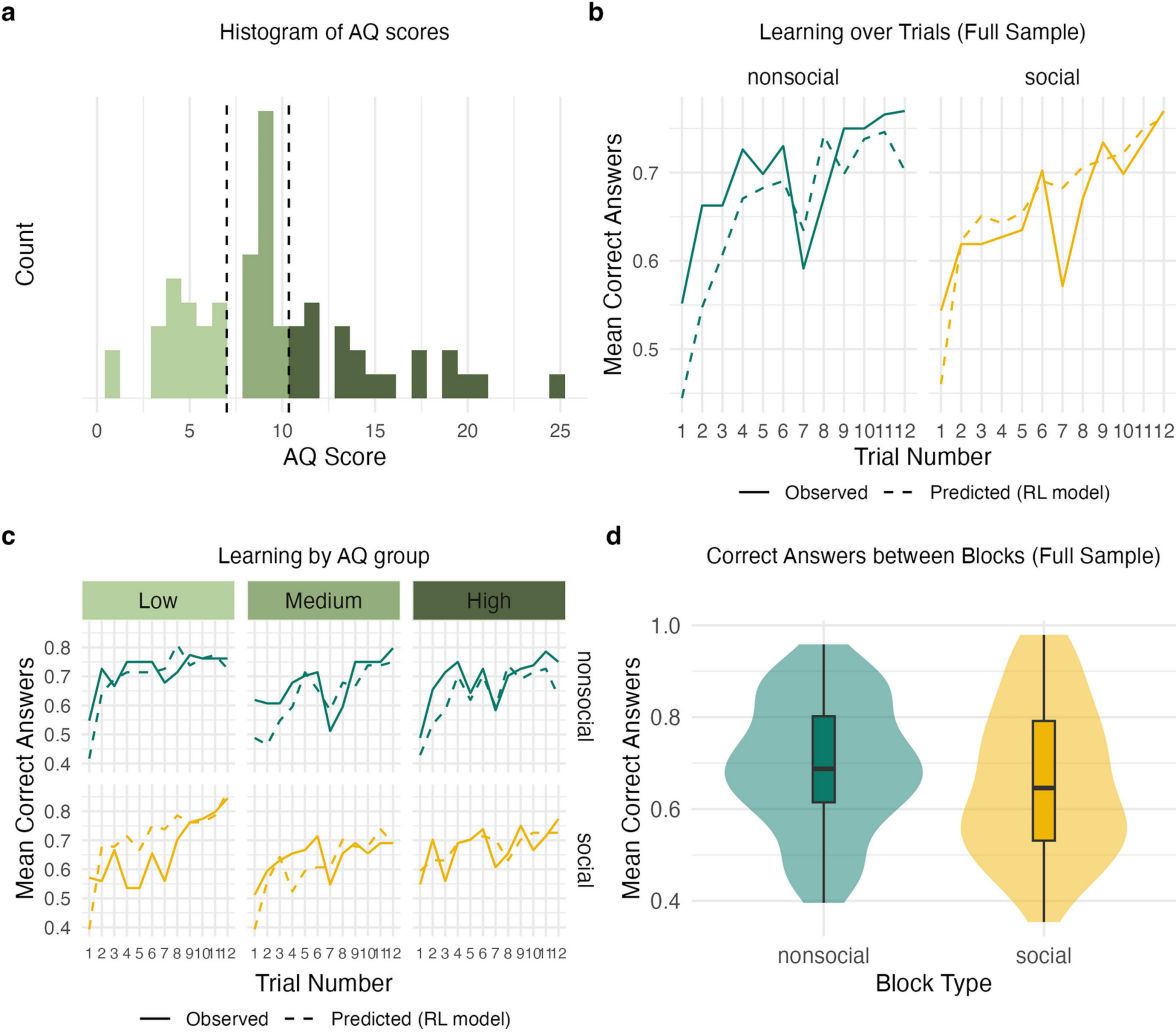
(a) Histogram of AQ scores, split into three groups indicated by dashed lines (low, medium, high). (b) Learning over trials for all participants. Trials 1−6 were presented in the first block, trials 7−12 in the second block, resulting in a delay and a drop in performance in trial 7. Predicted learning performance of the reinforcement learning (RL) model is depicted as dashed lines and indicates satisfactory model fit. (c) Learning over trials, split by three AQ groups depicted in the histogram (low, medium, high) for observed and predicted performance. Predicted performance of the RL model is depicted as dashed lines. (d) Violin plot and boxplots for correct answers between blocks, averaged over all trials. Performance was marginally lower in social blocks across the full sample, but not significantly (*p* = 0.23).

Sixty-three individuals were selected for analyses in the GLMM; 57.1% (*n* = 36) of the sample were females and 42.9% (*n* = 27) were male. The majority of the sample was composed of young adults (mean age = 26.51, s.d. = 6.76), with the oldest participant being 63 years old. With regard to AQ scores, the sample mean was *M* = 9.45, s.d. = 4.88 and values ranged from 1 to 25 (see figure 2a). Skewness of AQ scores was 0.82, indicating a moderately right-skewed distribution. This suggests that while most participants scored lower on the AQ, a smaller number of participants had higher scores, leading to a longer right tail.

### 3.2. Generalized linear mixed model

GLMM analyses revealed that trial number was significantly associated with higher odds of giving a correct answer OR = 1.10, 95% CI [1.06, 1.14], *p* < 0.001. This suggests that individuals learned the underlying association over trials, increasing their odds of giving a correct answer on average by 10% each trial. The main effect of block type (non-social versus social), was not significant, OR = 0.81, 95% CI [0.61, 1.09], *p* < 0.23, although learning performance was marginally lower in social blocks (as suggested by an OR value below one).

**Figure 3.**
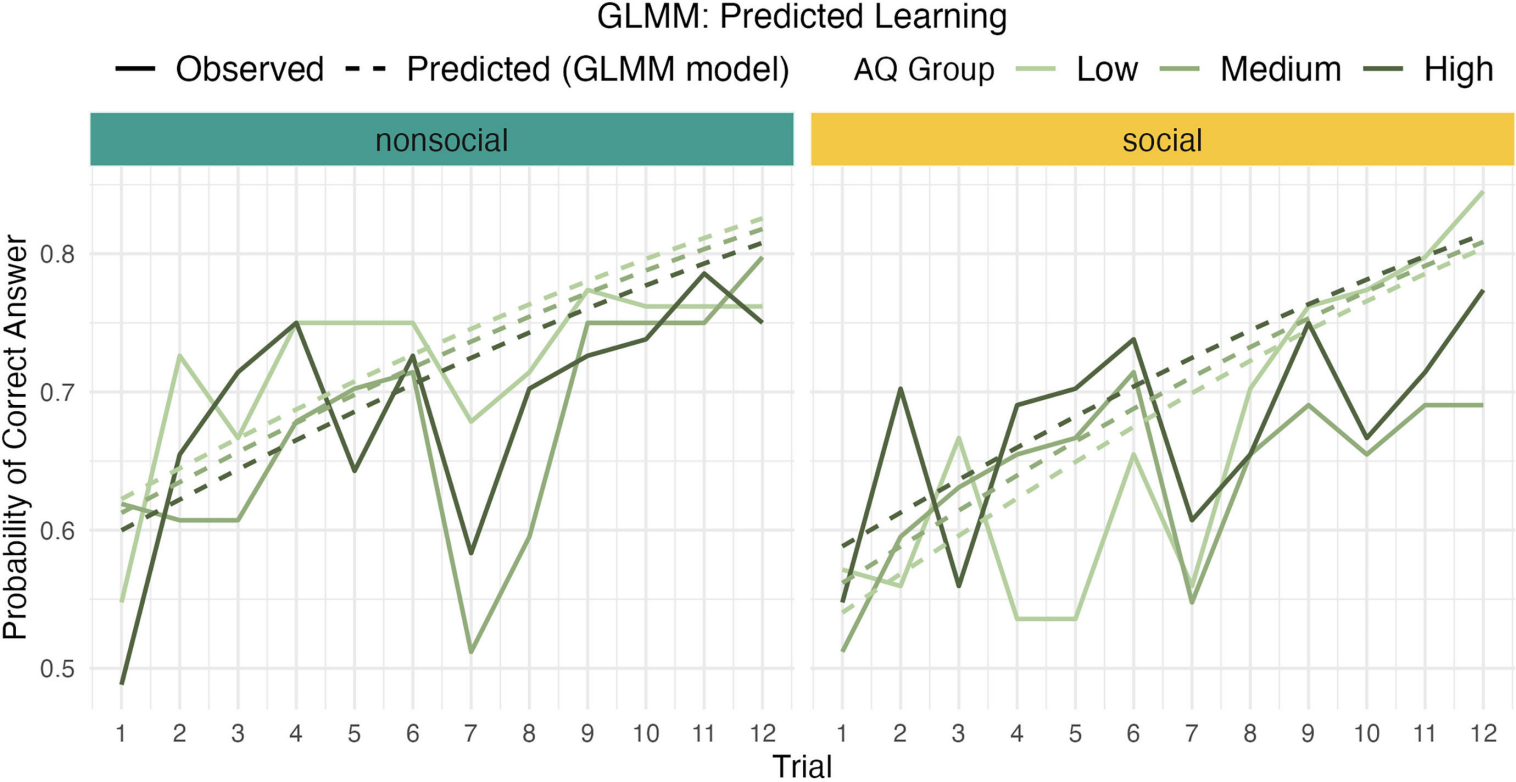
Observed (solid) and predicted (dashed) line plots, depicting learning over trials. Participants improve over trials, which is captured by the positive slope of model predictions. However, learning is not significantly different between block types or AQ groups (all *p* > 0.05).

The interaction between block type and AQ was not significant, OR = 1.03, 95% CI [0.97, 1.10], *p* = 0.22. This suggests that the effect of AQ did not differ significantly between block types. Similarly, the interaction between AQ score and trial number, the interaction between block type and trial number as well as the three-way interaction were not significant (all *p* > 0.05). This suggests that autistic traits did not explain variance in learning trajectories over trials, as indicated by largely parallel learning trajectories for different AQ groups (see figure 3).

### 3.3. Rescorla–Wagner results

**Figure 4.**
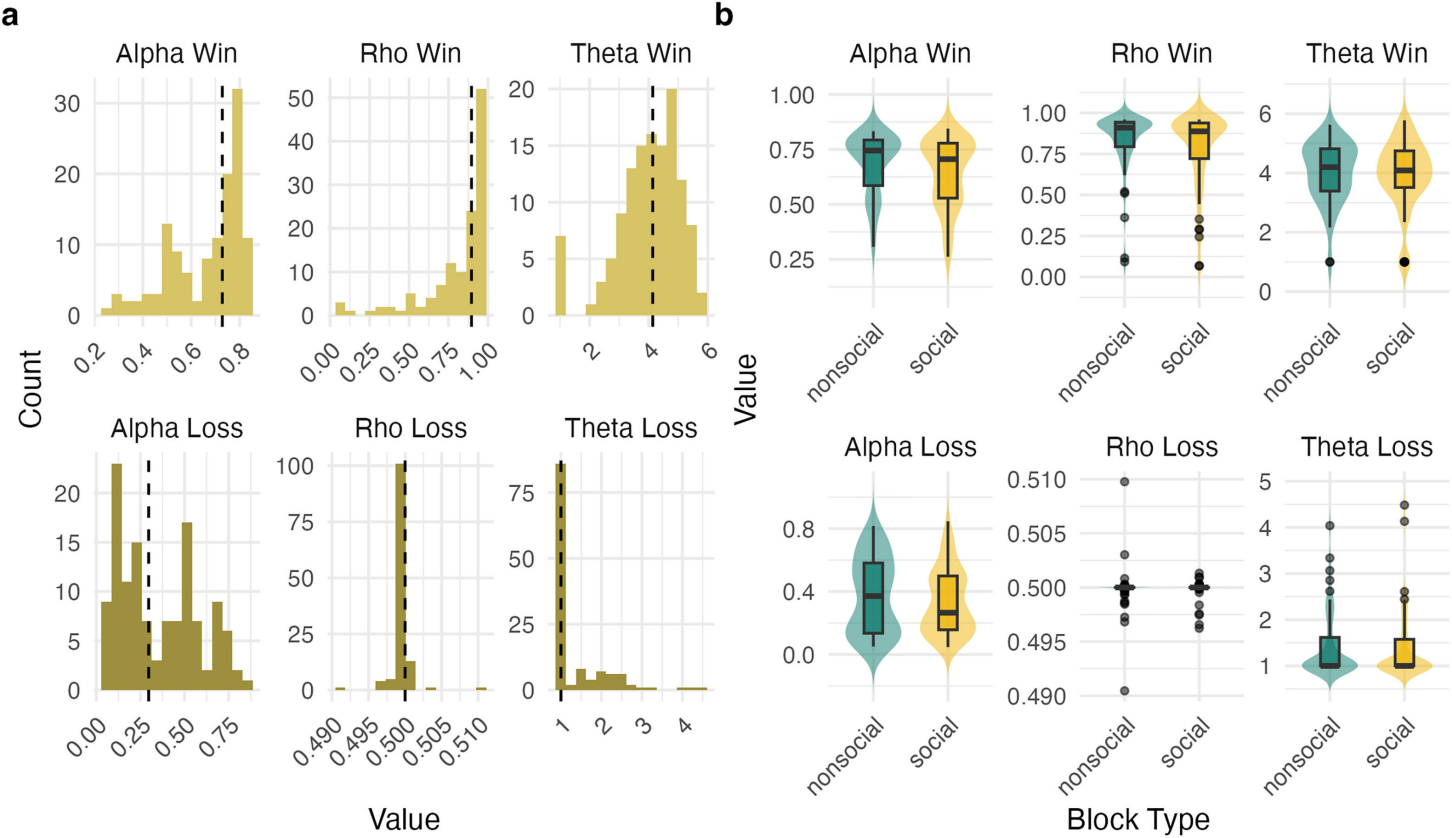
(a) Histograms of parameter distributions over all participants, separately for wins and losses. Dashed black lines depict median values. (b) Violin plots and boxplots for all parameters, comparing social and non-social blocks.

Model predictions and model fit for the estimated parameters can be investigated in figure 2b,c (dashed lines). Predictions follow the observed data closely, indicating satisfactory model fit. The histogram in figure 4a shows the distribution of fitted parameters over all participants. The non-parametric Wilcoxon signed-rank test suggested all parameters in the win domain to differ significantly from their counterparts in the loss domain (all *p* < 0.001), with parameters generally being larger for wins compared with losses.

When comparing parameters between social and non-social block types with random intercepts by subject, trends were observed for both *α*_win_ and *ρ*_win_ in a beta GLMM (see figure 4b). For *ρ*_win_, the difference between social and non-social blocks was close to significant, OR = 0.75, 95% CI [0.56, 1.01], *p* = 0.0596, suggesting *reward sensitivity* for wins was marginally lower in social blocks. For *α*_win_, the difference between block types was similar, OR = 0.83, 95% CI [0.68, 1.02], *p* = 0.0732, again suggesting that *learning rates* were marginally lower in social blocks. This aligns with the trend of reduced learning performance in social blocks described above; however, these differences failed to reach significance. For *θ*, no difference between block types was observed.

To assess the effect of AQ, a separate model with AQ and block type as predictors was fit for each parameter and domain (win/loss). For the beta-GLMMs, the main effect of AQ was significant for the *ρ*_win_ model, OR = 0.79, 95% CI [0.64, 0.98], *p* = 0.029 in a model specified without interaction. To further investigate the significance and robustness of this result, we performed two non-parametric tests, bootstrapping and a permutation test, both of which confirm the stability of the result, i.e. the empirical *p*-value derived from the permutation test was *p* = 0.003 (see electronic supplementary material, figure S3).

**Figure 5.**
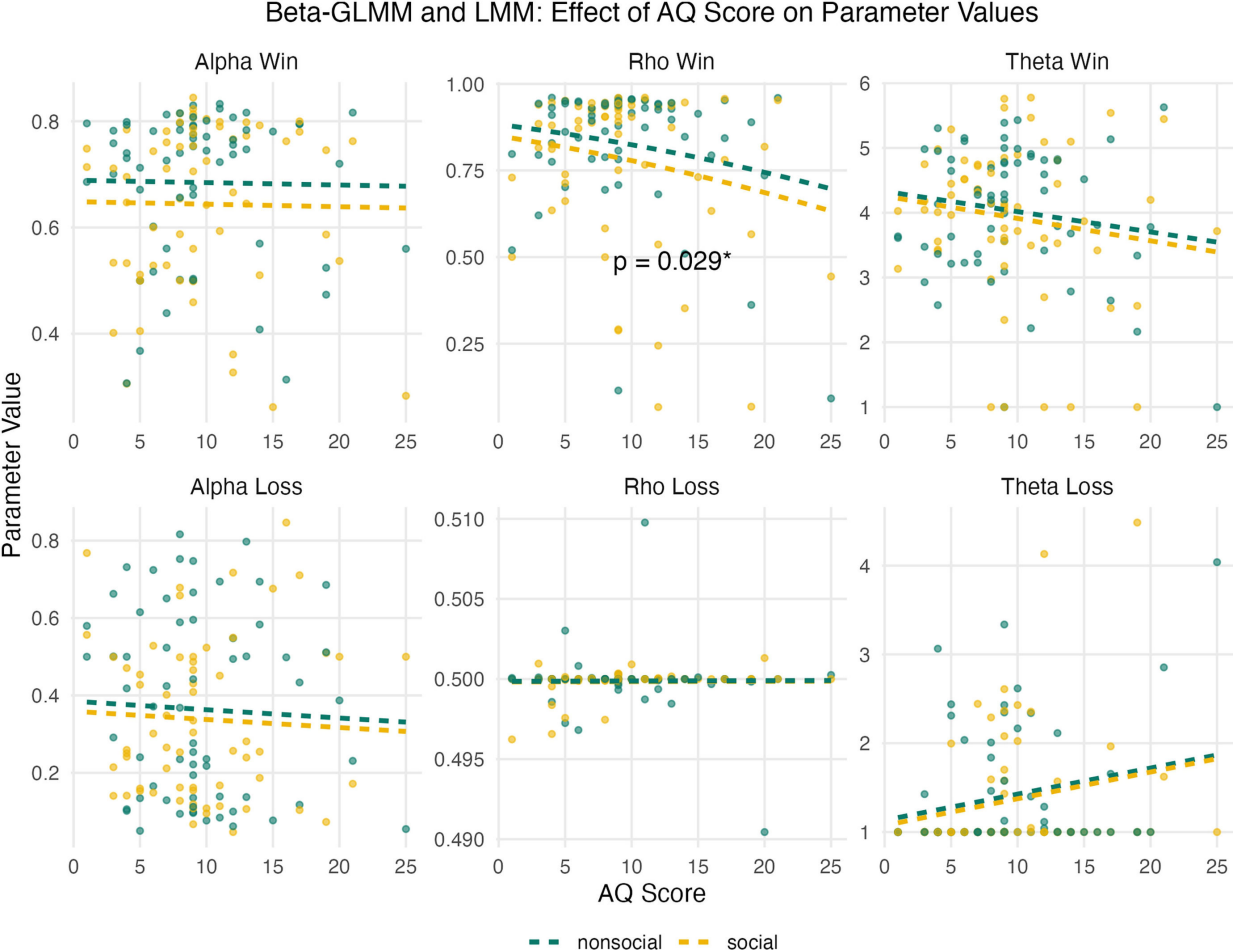
Scatterplots and line plots for the effect of autistic traits on parameter values in non-social and social blocks. Model predictions for each parameter are depicted as dashed lines. For alpha and rho values, a beta-GLMM was used, as parameters are bounded between 0 and 1. For theta values, an LMM was used, as parameter values are not bounded.

In a model with an interaction term, AQ × block type was not significant (*p* = 0.74), suggesting no difference in the relationship between AQ and *ρ*_win_ across social and non-social blocks. However, in a simple slope analysis, the effect of AQ was significant in social blocks, OR = 0.95, 95% CI [0.90, 0.999], *p* = 0.046, but not in non-social blocks, OR = 0.96, 95% CI [0.91, 1.01], *p* = 0.11. Importantly, while this finding suggests that higher AQ scores were associated with a decrease in *ρ*_win_ in the social condition, it does not provide evidence that the relationship differs significantly between social and non-social blocks, as indicated by the non-significant interaction term. Thus, caution should be exercised when interpreting differences between conditions. No main effects of AQ or interactions of AQ × block type were observed for *α*_win_ or *α*_loss_ (see figure 5, all *p* > 0.05).

*θ*_loss_, analysed with a linear mixed model, was significantly positively associated with AQ, *β* = 0.148, *t* = 2.23, *p* = 0.029, *r* = 0.27. This relationship is also evident in figure 5. However, as QQplots indicated that assumptions of linear models were violated for this model (see electronic supplementary material, figure S1), results should be interpreted cautiously. The interaction of block type and AQ was not significant for the *θ*_loss_ model (*p* > 0.5).

## 4. Discussion

To our knowledge, this study represents the first attempt to investigate the impact of autistic traits on learning from social and non-social PLDs matched on low-level visual properties. Our goal was to investigate raw learning performance, while also examining more subtle differences in the underlying learning processes by exploring parameters of a reinforcement learning model. These parameters were used to test competing theoretical accounts of ASD symptoms. In the analysis on raw learning performance, we did not detect significant differences related to autistic traits; however, computational modelling did reveal attenuated reward sensitivity in response to wins for participants with high autistic traits. We will first discuss the analysis of raw learning performance and then move on to more intricate observations derived from the reinforcement learning model.

A critical result in the first analysis is that participants’ performance improved over time in both social and non-social conditions, as evidenced by the significant effect of trial number. This illustrates that PLDs can successfully be used to investigate learning from social and non-social cues, and emotional expressions depicted in facial PLDs have been recognized to an extent that supports learning. Regarding the main effect of condition (social versus non-social), learning performance was marginally lower in the social condition across the entire sample, but this difference was not significant. This would indicate that learning from social PLDs was slightly harder, possibly due to higher ambiguity in the social condition (i.e. non-social PLDs could easily be interpreted, whereas facial PLDs required recognizing depicted emotions from point-light arrangements). However, this ambiguity did not significantly impact learning performance across the entire sample.

Regarding the effect of autistic traits, the main effect of AQ on learning performance was not significant, a result that is inconsistent with a general learning deficit for participants with high AQ, which would have manifested in decreased learning performance in both conditions. Contrary to our expectations, we also did not observe a significant interaction between condition and autistic traits, indicating that participants with high AQ scores did not perform worse in the social condition. As an explanation for the absence of this expected effect, we offer the following interpretation: participants with high AQ may have used *compensatory strategies* which did not rely on automatic emotion recognition, but rather on explicit cognitive effort. This compensatory strategy has been discussed in the literature as a potentially confounding factor in studies on facial emotion recognition and seems especially relevant in an adult sample of neurotypical individuals without ASD diagnosis [48]. Furthermore, emotional expressions in facial PLDs were mostly discernible by focusing on the mouth, whereas less emotional information was conveyed by the eyes. Deficits in emotion recognition in ASD have been shown to relate to fixations to the eye region [49], thus this feature of the stimuli may have contributed to the missing effect. Furthermore, debriefing information suggested that participants experienced emotion recognition in social PLDs as particularly challenging. Reliance on abstract visual stimuli thus may have led to increased difficulty in the social condition for participants with low AQ and enhanced learning performance for those with high AQ. Altogether, this might have potentially masked interindividual learning differences.

Despite the absence of differences in raw learning performance, computational modelling was able to uncover more subtle differences in the learning process. In particular, the analysis using the Rescorla–Wagner model showed a trend for a main effect of condition on both *reward sensitivity* and *learning rate* parameters for the win domain (*ρ*_win_ and *α*_win_). Both parameters were marginally lower in the social compared with the non-social condition over the entire sample, a finding that aligns with slightly lower learning performance in the social condition discussed above. Importantly, a significant main effect of AQ on *ρ*_win_ was observed, suggesting attenuated sensitivity for rewarding feedback in participants with higher autistic traits. This suggests that the *internal value* assigned to rewarding feedback was lower in participants with high AQ.

We did not observe a main effect of AQ on learning rates, again suggesting no general learning deficit over conditions. We also did not observe an interaction of AQ and condition on learning rates, i.e. no differences in learning rates related to AQ between social and non-social conditions. Lastly, *θ*_loss_, a parameter which captures the tendency to explore choice options in response to losses, was positively associated with AQ scores. This would suggest that participants with higher autistic traits exhibited more deterministic choice behaviour and less exploration in response to losses. This finding can be interpreted to align with symptoms of repetitive behaviour and insistence on sameness [50]; however, assumptions for this analysis are probably not met (see electronic supplementary material, QQplot, figure S1).

In summary, computational modelling indicates that participants with high autistic traits did not differ in terms of the weighting of prediction errors (neither social nor non-social), but rather in the way the *internal value* of stimuli was represented during learning. This aligns with the slow updating hypothesis [7,8], which suggests that rather than excessively depending on prediction errors, autistic individuals integrate recent information more gradually. Instead of rapidly adjusting beliefs based on new evidence, they rely more on longer-term accumulated knowledge. This is consistent with previous findings showing diminished trial-by-trial adjustments in both perceptual and motor learning tasks [7,8]. By contrast to the volatility hypothesis [5], which predicts excessive sensitivity to change, our results suggest that high-AQ individuals are less responsive to new feedback, supporting the idea that autistic learning difficulties might stem from underweighting recent experience.

Our results provide less support for the social motivation hypothesis [9], which suggests that autistic traits are linked to reduced learning specifically in social contexts. While we observed lower reward sensitivity in high-AQ participants, this effect was not stronger in the social condition. This suggests that autistic traits may affect learning more generally, rather than being driven by a diminished sensitivity to social rewards.

Some limitations of this study also need to be taken into account. First, observed differences in parameter values did not reflect in learning performance, i.e. we did not observe reduced learning for participants with high autistic traits. Thus, the effect of autistic traits on learning from PLDs was confined to the parameters of the reinforcement learning model. One could question whether a difference not reflected in learning performance is valid. However, we argue that differences in how associations are learnt, rather than exclusively focusing on learning outcome, is crucial for gaining a full picture of learning deficits in ASD. As mentioned above, compensatory mechanisms could have been employed by participants with high AQ to arrive at the same level of performance; however, the process might differ.

Second, a potential confound in this study is the role of working memory [51]. The task required participants to hold and manipulate information about probabilistic feedback, which places a significant demand on working memory resources. Differences in working memory capacity could influence participants' performance independently of their learning rates or reward sensitivities. We did not record measures of working memory in this study; however, we screened for intelligence deficits and did not include individuals with reduced cognitive abilities. Furthermore, as discussed above, a direct effect of AQ on learning performance was not observed. Thus, it seems unlikely that working memory would be a confound in these analyses. Still, future studies should consider assessing and controlling for working memory capacity to disentangle its effects from those of the primary variables of interest.

Lastly, it is important to note that the study did not include participants with ASD but neurotypical controls from the healthy population. Although many findings on learning from social stimuli generated in the ASD population have been shown to generalize to autistic traits in neurotypicals [23–28], this limitation should be considered when extending our findings to individuals with ASD.

## 5. Conclusions

Despite limitations mentioned above, this study provides an important addition to the field of reinforcement learning in ASD, constituting the first attempt to employ carefully matched PLD stimuli in a reinforcement learning task. Results illustrate significant effects of autistic traits on different parameters of the learning process, providing a detailed description of learning deficits and their relation to theoretical models of ASD. Specifically, high autistic traits were related to reduced reward sensitivity in the win domain. By contrast, no effects of autistic traits were observed for learning rates, demonstrating the importance of distinguishing between different parameters of computational models. Future studies aiming to unravel distinctive contributions of learning rates and reward sensitivities could apply reinforcement learning from PLDs in conjunction with functional magnetic resonance imaging (fMRI): PLDs are especially useful in situations where visual properties of the stimulus need to be fully accounted for. Activation in areas linked to face perception such as the fusiform face area (FFA) [36] or dissociable components of motivation, such as the ventral striatum and the ventromedial prefrontal cortex [52] could then be related to perception of facial PLDs and model parameters. This could give further insight into social learning deficits in ASD and shed light on the root causes of observed social deficits.

## Data Availability

Data and relevant code used in the analysis are available in a Dryad and an associated Zenodo repository: [53,54]. A version of the task that runs in the browser is available on github (Chrome recommended): https://raimund-buehler.github.io/SOCIALRL_PLD/. The code for creating the task has also been archived within a separate Zenodo repository [55]. Supplementary material is available online at [56].
